# Propensity for COVID-19 severe epidemic among the populations of the neighborhoods of Fortaleza, Brazil, in 2020

**DOI:** 10.1186/s12889-020-09558-9

**Published:** 2020-10-01

**Authors:** Jose Ueleres Braga, Alberto Novaes Ramos, Anderson Fuentes Ferreira, Victor Macêdo Lacerda, Renan Monteiro Carioca Freire, Bruno Vieira Bertoncini

**Affiliations:** 1 Department of Epidemiology, Institute of Social Medicine, State University of Rio de Janeiro, Rua São Francisco Xavier 524 / 7017D, 7° floor, Rio de Janeiro, RJ CEP 20550-013 Brazil; 2 Department of Epidemiology and Quantitative Methods, Sergio Arouca National School of Public Health (ENSP), Oswaldo Cruz Foundation (FIOCRUZ), Rio de Janeiro, RJ Brazil; 3 Department of Community Health, School of Medicine, Federal University of Ceará (UFC), Fortaleza, Brazil; 4 Postgraduate Program in Public Health, School of Medicine, Federal University of Ceará (UFC), Fortaleza, Brazil; 5 Municipal Secretariat of Conservation and Public Services (SCSP), Fortaleza, Brazil; 6 Department of Transport Engineering, Technology Centre, Federal University of Ceará (UFC), Fortaleza, Brazil; 7 Postgraduate Program in Transport Engineering, Technology Centre, Federal University of Ceará (UFC), Fortaleza, Brazil

**Keywords:** COVID-19, Public health surveillance, Disease outbreaks

## Abstract

**Background:**

The state of Ceará (Northeast Brazil) has shown a high incidence of coronavirus disease (COVID-19), and most of the cases that were diagnosed during the epidemic originated from the capital Fortaleza. Monitoring the dynamics of the COVID-19 epidemic is of strategic importance and requires the use of sensitive tools for epidemiological surveillance, including consistent analyses that allow the recognition of areas with a greater propensity for increased severity throughout the cycle of the epidemic. This study aims to classify neighborhoods in the city of Fortaleza according to their propensity for a severe epidemic of COVID-19 in 2020.

**Methods:**

We conducted an ecological study within the geographical area of the 119 neighborhoods located in the city of Fortaleza. To define the main transmission networks (infection chains), we assumed that the spatial diffusion of the COVID-19 epidemic was influenced by population mobility. To measure the propensity for a severe epidemic, we calculated the infectivity burden (I_ty_B), infection burden (I_on_B), and population epidemic vulnerability index (PEVI). The propensity score for a severe epidemic in the neighborhoods of the city of Fortaleza was estimated by combining the I_on_B and PEVI.

**Results:**

The neighborhoods with the highest propensity for a severe COVID-19 epidemic were Aldeota, Cais do Porto, Centro, Edson Queiroz, Vicente Pinzon, Jose de Alencar, Presidente Kennedy, Papicu, Vila Velha, Antonio Bezerra, and Cambeba. Importantly, we found that the propensity for a COVID-19 epidemic was high in areas with differing socioeconomic profiles. These areas include a very poor neighborhood situated on the western border of the city (Vila Velha), neighborhoods characterized by a large number of subnormal agglomerates in the Cais do Porto region (Vicente Pinzon), and those located in the oldest central area of the city, where despite the wealth, low-income groups have remained (Aldeota and the adjacent Edson Queiroz).

**Conclusion:**

Although measures against COVID-19 should be applied to the entire municipality of Fortaleza, the classification of neighborhoods generated through this study can help improve the specificity and efficiency of these measures.

## Background

The first case of coronavirus disease (COVID-19) was recorded on December 8, 2019, in Wuhan, Hubei province, China. Approximately 3 months later, on March 11, 2020, this disease was declared as a pandemic by the World Health Organization (WHO) [1]. On February 3, 2020, the Brazilian Ministry of Health declared COVID-19 as a Public Health Emergency of National Importance (ESPIN), through Ordinance MS n. 188 of 2020, in line with Decree n. 7616, of November 17, 2011.

The first confirmed COVID-19 case in Latin America was reported on February 26, 2020, in the city of São Paulo, the most populous city in the Southern Hemisphere (approximately 12 million inhabitants), and involved a person who had travelled to northern Italy (Lombardia region) [2, 3]. Approximately a month later, on March 20, Ordinance MS n. 454 was published in the Official Gazette (Edition: 55-F / Section 1 - Extra), declaring the existence of community transmission of COVID-19 throughout the national territory of Brazil. Since then, Brazil has registered the largest number of confirmed cases in Latin America (*n* = 20,727, 18:00, April 11, 2020) [4].

Ceará has become one of the states with the highest number of cases of the disease (1374), with only São Paulo and Rio de Janeiro reporting higher numbers (6708 and 1938, respectively). While the average incidence rate in Brazil is 7.5 cases per 100,000 inhabitants, the rate in Ceará is double at 14.1 per 100,000 inhabitants [5]. The highest number of cases and deaths is concentrated in Fortaleza (1291 confirmed cases and 53 deaths), making it the Brazilian state capital with the highest incidence rate (35/100,000), and a mortality rate of 3.6% among confirmed cases [4].

Numerous factors have been considered important to explain the magnitude, intensity, and early viral circulation of the epidemic in Fortaleza. Of these, the following two aspects appear to be the most crucial: firstly, the Pinto Martins International Airport in Fortaleza, which became a “flight hub” in 2018, facilitated the influx of a large number of national and international flights and tourists; and secondly, the measures adopted by the state epidemiological surveillance services included testing a relatively large number of suspected cases.

Monitoring the dynamics of the COVID-19 epidemic in Fortaleza is of strategic importance for reducing overall epidemic burden and requires the use of sensitive tools for epidemiological surveillance, including analyses that allow the recognition of areas with a greater propensity for increased severity throughout the epidemic cycle. In addition to their technical-scientific character, these analyses must be strongly integrated with operational aspects of surveillance and control, with cooperation of public managers of the municipality in the health, transport, and education sectors, to achieve timely and effective integrated responses like allocation of resources and medical personnel depending on determined propensity of severe epidemic.

The study proposes to increase the understanding of the COVID-19 epidemic in Fortaleza, enabling the recognition of urban spaces with a greater propensity for a severe epidemic based on population mobility in order to promote evidence-based actions by the municipality public authorities. This study aims to classify neighborhoods in the city of Fortaleza by their propensity for a severe COVID-19 epidemic in 2020.

## Methods

### Study design

**Fig. 1:**
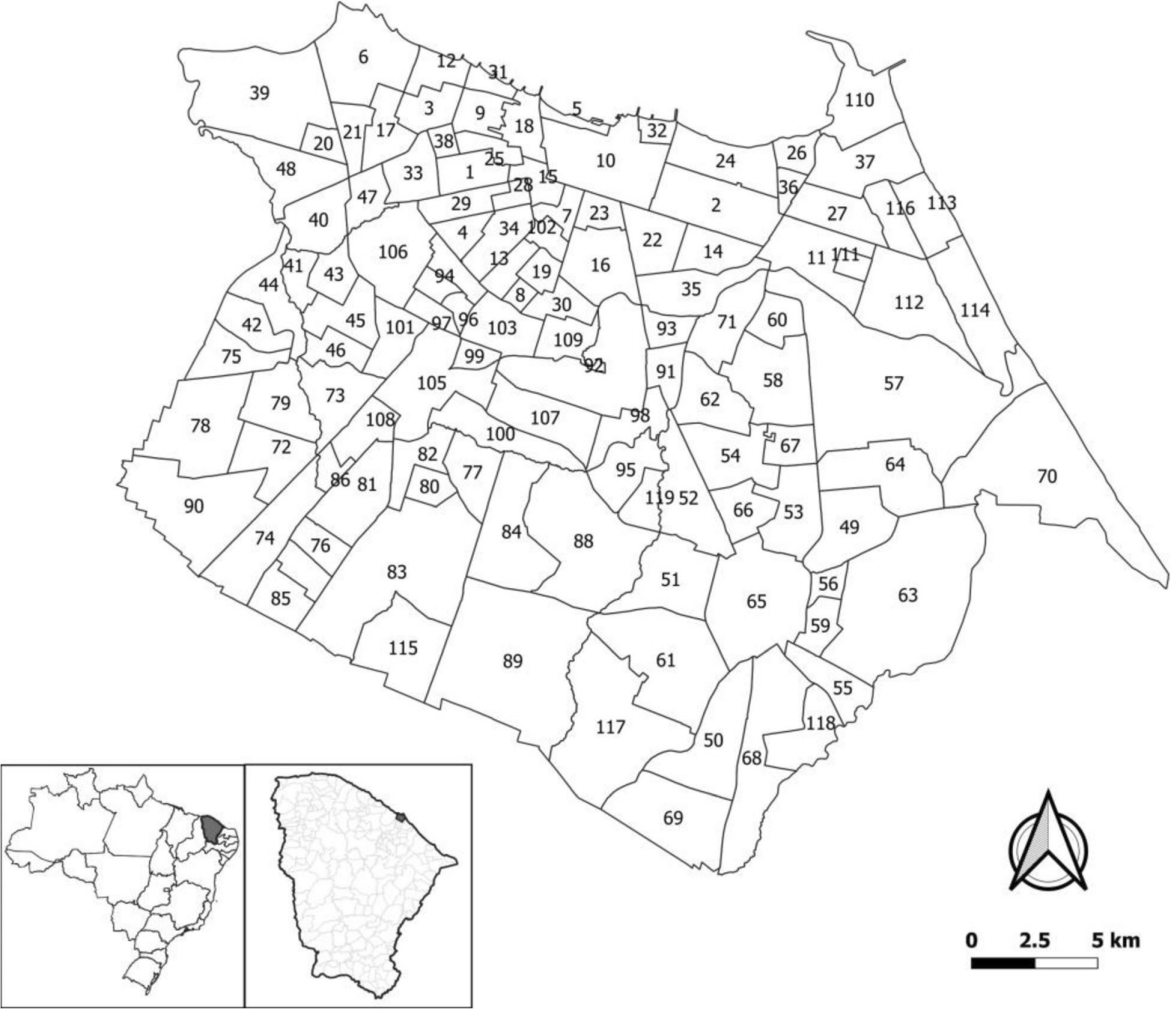
Map of the 119 neighborhoods in the municipality of Fortaleza

This is an ecologic study based on the geographical unit of the 119 neighborhoods in the municipality of Fortaleza (Fig. 1). In order to define the main transmission networks (and chains), we hypothesized that the spatial diffusion of the COVID-19 epidemic is influenced by population mobility. We also postulated that the spatial spread of the COVID-19 epidemic follows the hierarchical model based on networks and population mobility that plays a key role in the constitution of the viral transmission chains.

The rationale of the study assumed that based on the incidence rate of the initial phase of the epidemic (infectivity load), the flow of passengers moving between neighborhoods can influence the infection load in each neighborhood of the municipality. If one considers the epidemic vulnerability attributed to populations living in these neighborhoods, combined with the mentioned infection burden, one can estimate the propensity for a serious COVID-19 epidemic.

To measure the propensity of a severe epidemic, the following events were initially calculated: (*i*) infectivity burden - I_ty_B; (*ii*) infection burden - I_on_B; and (*iii*) population epidemic vulnerability index (PEVI). Then, the (*iv*) Propensity score for a severe epidemic in the neighborhoods of the city of Fortaleza was estimated.

### Infectivity burden

Infectivity burden was calculated using the epidemiological surveillance records of COVID-19 in Fortaleza, so the number of confirmed cases were denoted as infectivity burden. These data were formally obtained from the Municipal Health Department on April 7, 2020. The suspected cases of COVID-19 had been investigated according to the recommendations of the Ministry of Health. All confirmed COVID-19 cases with SARS-CoV-2 PCR positive status reported to the Municipal Health Department of Fortaleza until March 12, 2020, residing in the municipality, were included. This period corresponds to the initial phase of the epidemic, when predominantly imported cases (travelers) initiated the transmission; indigenous cases would be detected later after being in contact with the initial cases through community transmission [6]. In this phase, the Fortaleza City Council adopted more general measures for surveillance, prevention, and control of COVID-19 [7].

### Infection burden

The Infection burden was measured by combining the infectivity burden and population mobility between the neighborhoods of Fortaleza. The mobility burden - M_ty_B, was evaluated through the daily travel flows, by looking at public transport use with work motivation between the two neighborhoods. Notably, the measurement of the flow of people between the neighborhoods of Fortaleza (excluding displacements within the same neighborhood) only became possible after a strategic study was conducted by the city of Fortaleza and other institutions on this theme in 2019 called Home Origin-Destination Survey (OD survey).

The OD survey comprised a sample survey performed through interviews in households, providing the values of the variables in this analysis with a detailed matrix of the trip patterns and travel choices. Data were collected in a database, which describes the various attributes of the activities and trips of the city’s inhabitants, as well as the respective socioeconomic status and characteristics of individuals and their families, in order to describe a pattern of displacement of people and the chain of their activities throughout a typical business day. The information from the OD Matrix is of immense importance in the analysis of transport systems, comprising fundamental elements for planning and decision making, and has therefore been integrated in this study. The infection burden indicator was calculated using the following formula:

$$ {I}_{on}{B}_i=\sum \limits_{j=1}^{118}{I}_{ty}B\ast {M}_{ty}B $$

### PEVI

The PEVI was constructed according to the Urban Health Index approach recommended by WHO [8], to demonstrate the population attributes that best represent, from a collective point of view, the vulnerability of the population to COVID-19. This index comprises seven (7) sociodemographic indicators, based on the 2010 Brazilian census of the Brazilian Institute of Geography and Statistics (IBGE). The indicators that make-up the PEVI are: (*i*) proportion of households with more than two residents per bedroom, (*ii*) illiteracy proportion, (*iii*) proportion of the population in extreme poverty, (*iv*) proportion of households without running water and sanitation, (*v*) proportion of unemployment, (*vi*) Gini of family income, and (*vii*) proportion of people living in subnormal agglomerations.

IBGE classifies subnormal agglomerates groups as consisting of 51 or more housing units, characterized by the absence of ownership titles, and at least one of the following characteristics: irregularity of circulation routes, size and shape of the lots, and lack of essential public services (such as garbage collection, sewage, water, electricity, and public lighting).

The data for each of the indicators above were previously obtained and translated into the Human Development Units (HDUs) by the Institute for Applied Economic Research (IPEA), with the exception of the proportion of subnormal agglomerations. The HDUs represent units of analysis with relatively homogeneous socioeconomic characteristics, and the original data were used to produce the Metropolitan Region Human Development Atlas. The HDUs were designed to generate more homogeneous areas, based on socioeconomic conditions, than the weighted areas of the IBGE. To calculate the proportion of subnormal agglomerations, data from the IBGE were used, considering the population living under these conditions by the total population of the neighborhood, thereby obtaining the percentage of people living in subnormal agglomerations per neighborhood.

After the values of the indicators were stored in a database, the accuracy, completeness, and consistency of the data were verified for calculation of the summary measure. There are two main steps in calculating the index: (1) standardization of indicators, and (2) amalgamation of standardized indicators. Each of these steps can be performed in a mathematically straightforward manner.

The standardization of the values of each indicator is performed using the following formula:

$$ {I}^p=\frac{I-\mathit{\min}(I)\ }{\mathit{\max}(I)-\mathit{\min}(I)} $$

where *I*^*p*^ is the standardized value of I, max (I) is the highest value of I among all observations, and min (I) is the lowest value of I among all observations.

Since the *I*^*p*^ values are obtained for all indicators and units, the next step was to integrate *I*^*p*^ into a single composite index, here called VEPI. VEPI is calculated for each unit, using the geometric mean of the *I*^*p*^ values for each unit. Considering that there are j indicators, the formula used for this calculation was:

$$ VEPI={\left(\prod \limits_{i=1}^j{I}_i^p\right)}^{\frac{1}{j}} $$

Where, ${\mathrm{I}}_i^p$ is the standardized value of the seven indicators for a given neighborhood, and j corresponds to all other neighborhoods.

**Fig. 2:**
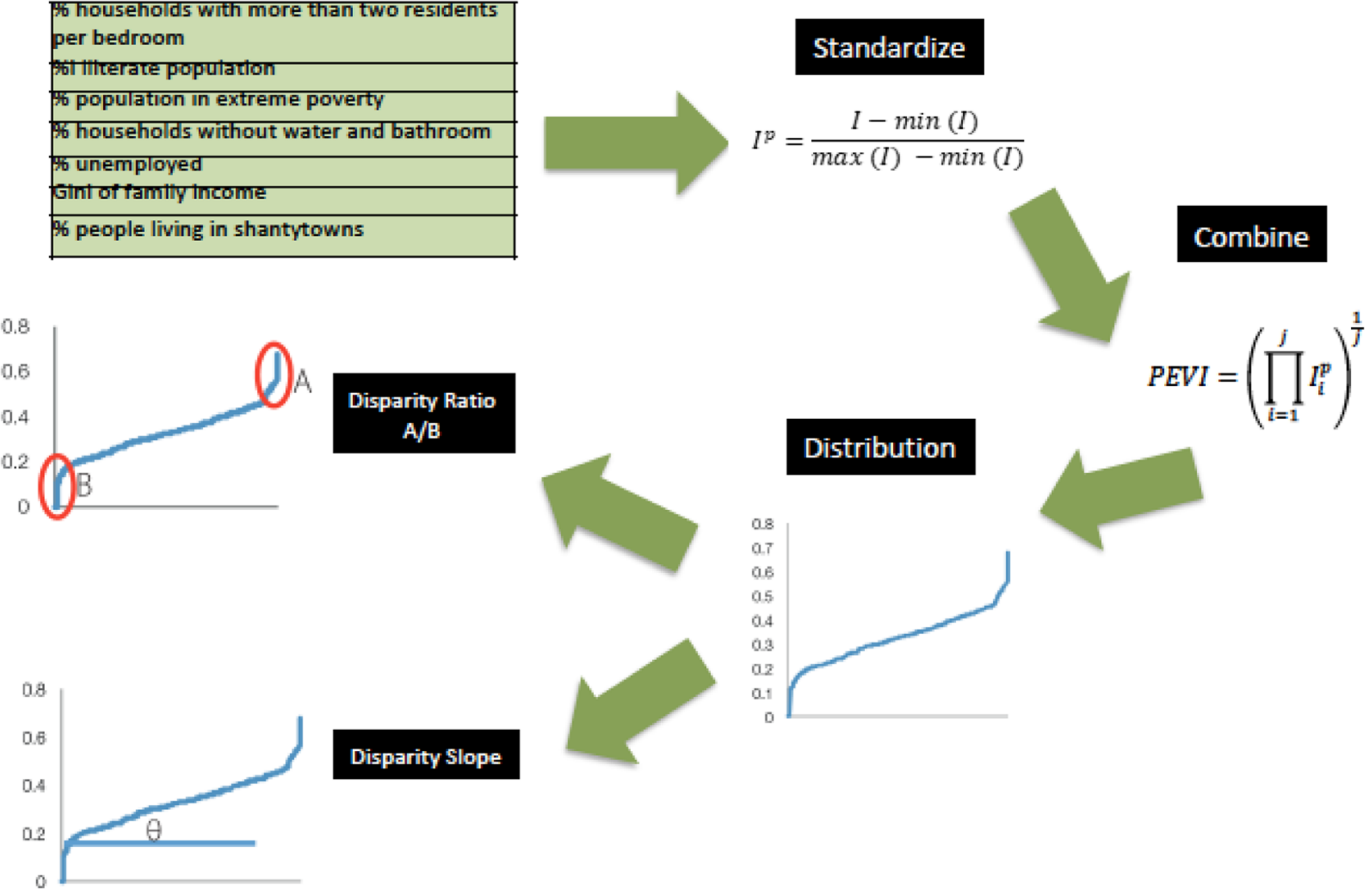
Flow Diagram for constructing the population epidemic vulnerability index

In addition to the point estimate, VEPI variance and standard error were also calculated, with respective confidence interval estimates based on these measures. Considering that the purpose of this index is to identify the “geographical” disparities of the studied phenomenon, a diagnosis of its distribution, evaluating the differences between the highest and lowest values as well as its visual comparison with the homogeneous distribution of these values, was carried out through the “qqnorm” graph (Fig. 2).

### Severe epidemic population propensity

The main study outcome is the Severe Epidemic Population Propensity. The estimated score of the propensity for a severe epidemic amongst the population of the Fortaleza neighborhoods was calculated by combining the infection burden with PEVI. The calculation was carried out using the multiplicative approach, involving the multiplication of these two indexes.

## Results

During the initial phase of the epidemic described in this study, from February 27 to March 12, 2020, 54 cases of COVID-19 were detected and reported, and this was the scenario used to study the propensity for a severe epidemic of populations living in the neighborhoods of the municipality of Fortaleza.

**Fig. 3:**
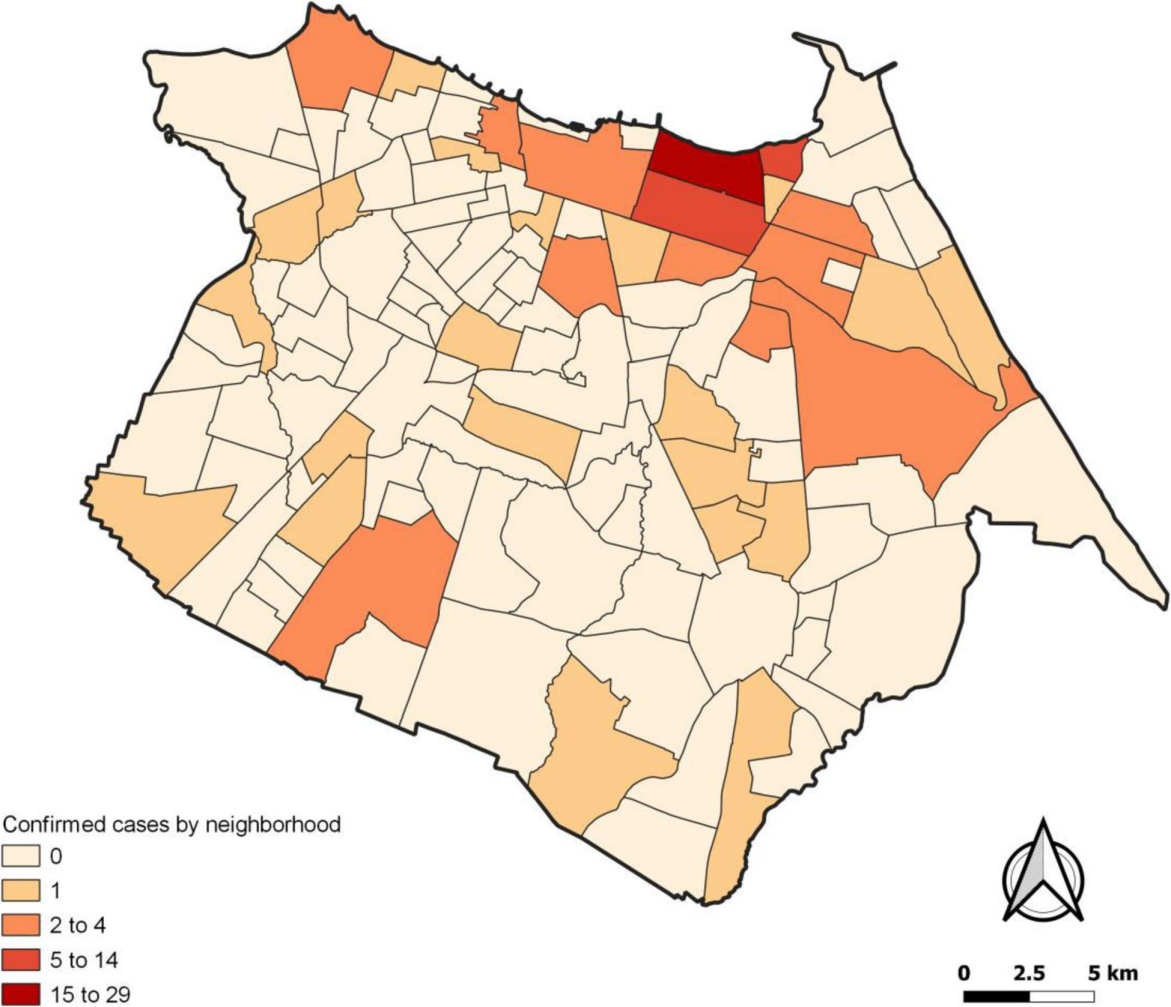
Map of the distribution of confirmed cases of COVID-19 in the neighborhoods of the Municipality of Fortaleza, from February 27 to March 12, 2020

The spatial distribution of COVID-19 incidence in this period indicates that the epidemic was restricted to specific areas of the city. This distribution reflects important heterogeneity, with a concentration of cases in the central area and in neighborhoods that represent better socioeconomic conditions, and also in areas with more diverse socioeconomic conditions, such as Meireles, Aldeota, Papicu, and Cocó (Fig. 3).

During the beginning of the epidemic, the spatial distribution of the infectivity burden by neighborhood reflects heterogeneity, concentrated mainly in central areas and in neighborhoods with the highest economic conditions. This pattern indicates, at this stage, a low burden of COVID-19 in the outskirts of the city. This distribution is, therefore, similar to the distribution of incidence, and compatible with the surveillance strategy adopted by the state and municipal governments for this first stage of the epidemic curve caused by COVID-19.

**Fig. 4:**
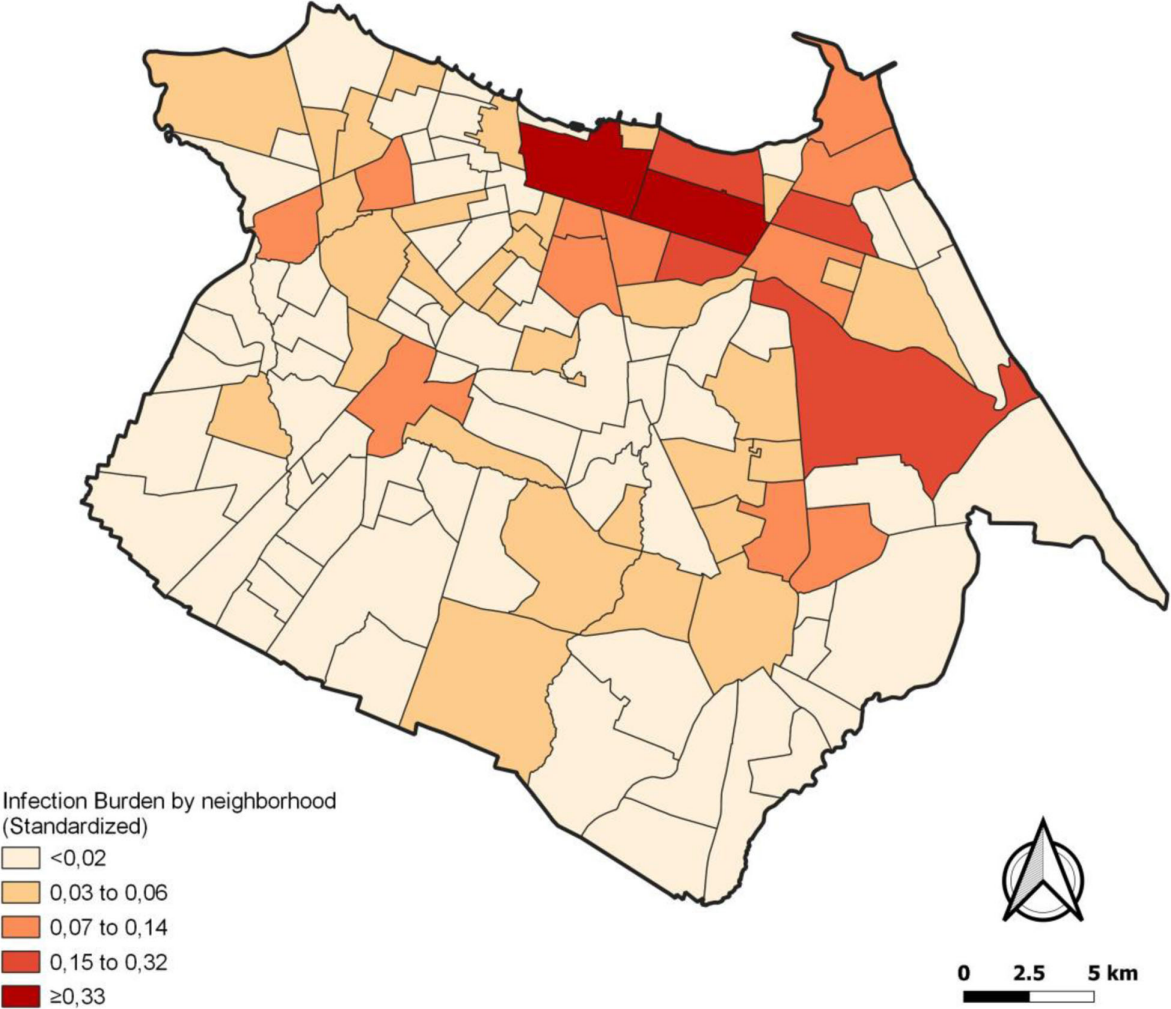
Map of the distribution of the COVID-19 infection burden in the neighborhoods of the Municipality of Fortaleza

The distribution of the infection burden (Fig. 4) was quite heterogeneous, with some areas showing higher levels than those of the central and eastern areas, as this measure reflects the combination of the infectivity burden and population mobility between the neighborhoods of Fortaleza.

**Fig. 5:**
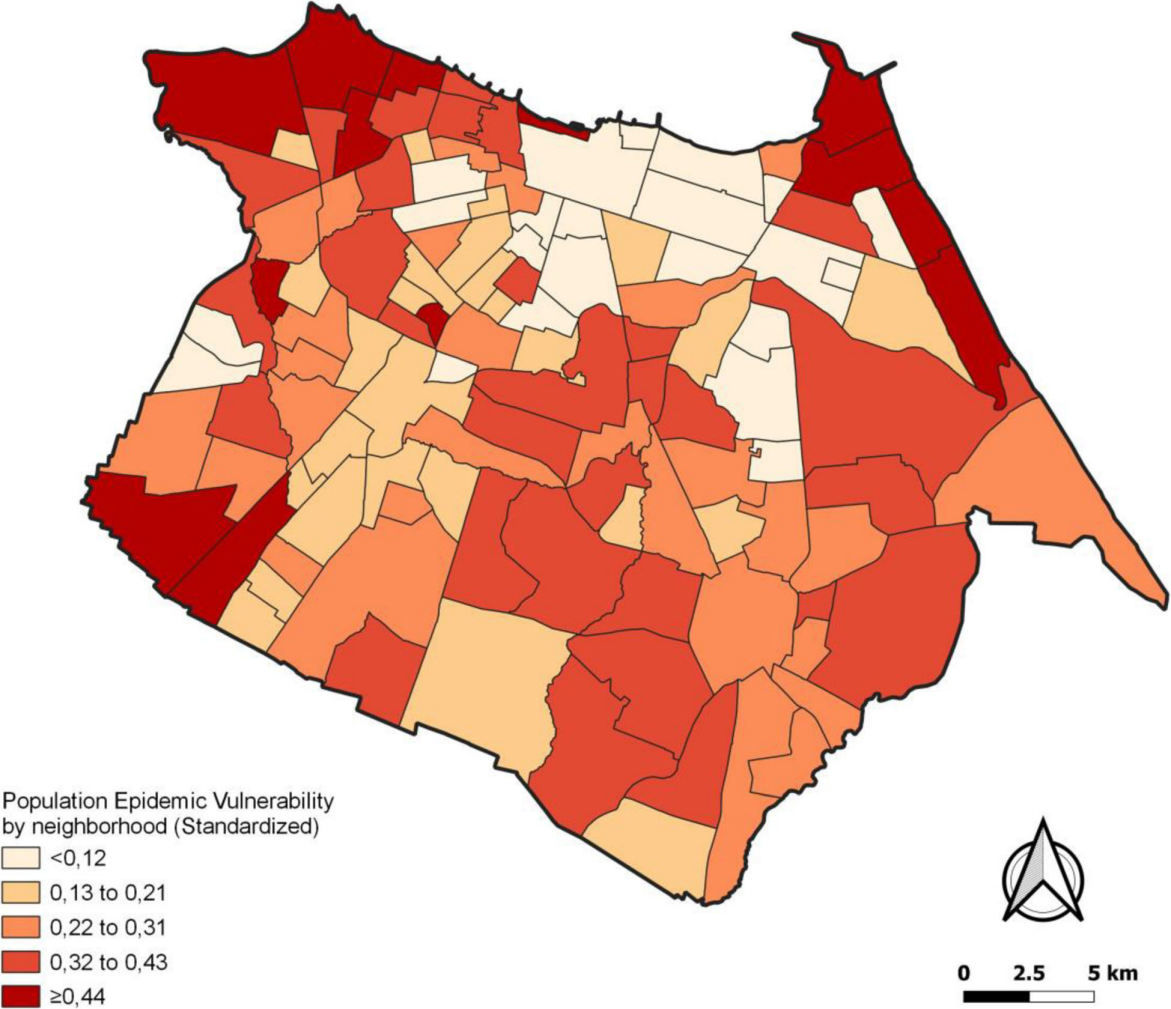
Map of the distribution of the population epidemic vulnerability to COVID-19 according to neighborhoods in the Municipality of Fortaleza

The VEPI index had a very heterogeneous spatial distribution, similar to the distribution of the seven indicators that were combined for its construction. The distribution shown in Fig. 5 indicates low vulnerability in the central and central-eastern regions of the city and increased levels of vulnerability as the neighborhoods move away from this region. However, it can be observed that some areas of the southern periphery, the western zone, and the eastern coastal zone have the highest levels of vulnerability.

When looking at the distribution of vulnerability to the COVID-19 epidemic, the combination of the various attributes related to the seven indicators appears to reveal something different than the individual indicators. An example is the vulnerability of the neighborhoods in the Cais do Porto region, the eastern coastal zone, and the eastern part of the city, which seemed to have been mainly influenced by the existence of subnormal agglomerations.

**Fig. 6:**
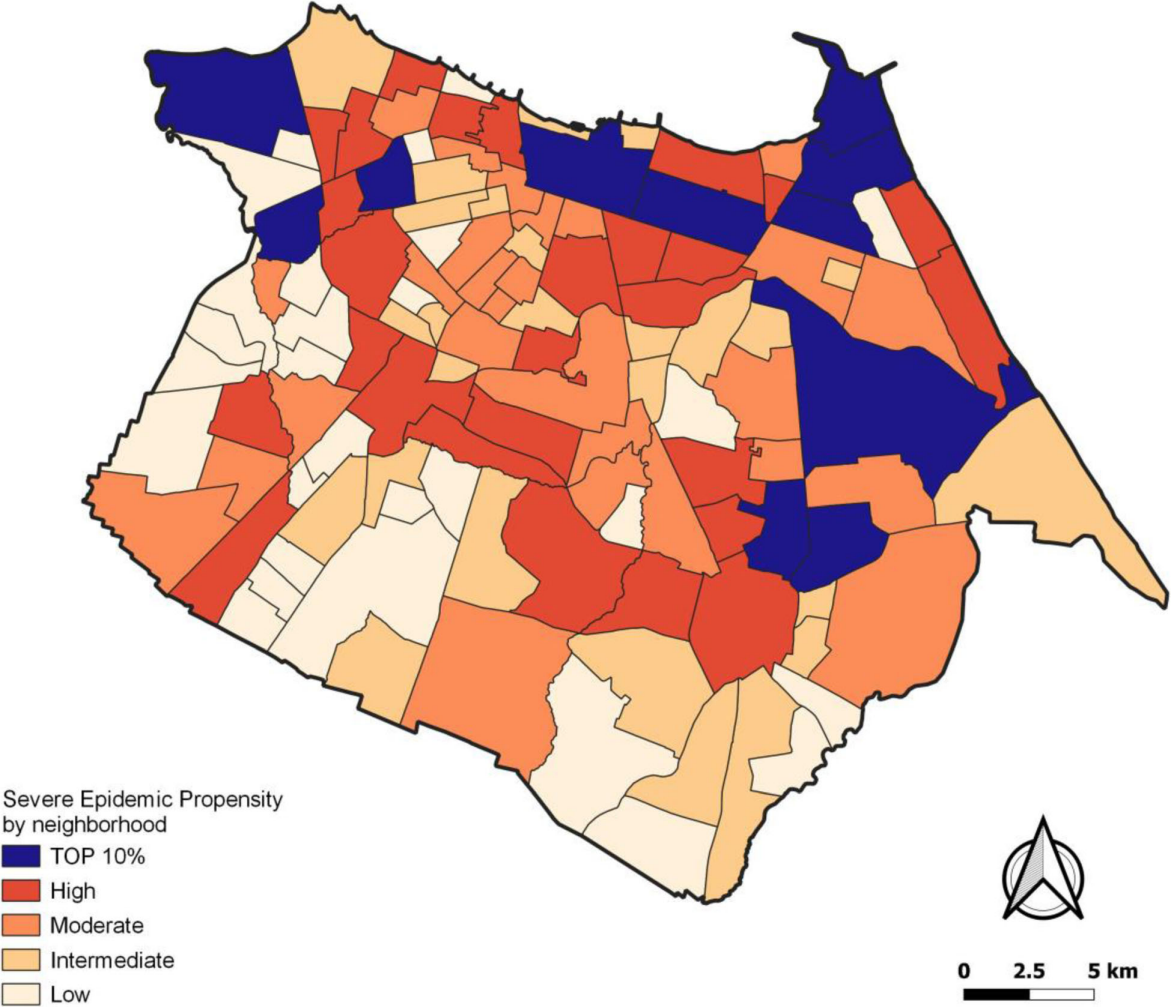
Map of COVID-19 severe epidemic propensity according to neighborhoods in the city of Fortaleza

The distribution of the propensity shown in Fig. 6 indicates that, besides being heterogeneous, the detected weaknesses (epidemic vulnerability) combined with the initial situation of the COVID-19 epidemic (period until March 12, 2020) and the population mobility lead to the prediction that neighborhoods such as Centro, Aldeota, and Papicu are among those in the top 10% with the highest propensity score. Other neighborhoods are also classified in the same group (western zone, bordered by Caucaia). These results are not as evident when the various aspects that influence the epidemic are evaluated separately.

## Discussion

The neighborhoods with the highest propensity for a severe COVID-19 epidemic were Aldeota, Cais do Porto, Centro, Edson Queiroz, Vicente Pinzon, Jose de Alencar, Presidente Kennedy, Papicu, Vila Velha, Antonio Bezerra, and Cambeba. The main finding of this study indicates higher levels of propensity to the COVID-19 epidemic in areas with a wide spectrum of socioeconomic profiles, including a group of very poor neighborhoods on the western border of the city (Vila Velha), a set of neighborhoods characterized by a large number of subnormal agglomerates in the Cais do Porto region (Vicente Pizon), and neighborhoods in the oldest central area of the city, where low-income areas exist despite the overall wealth in the area (Aldeota and the adjacent Edson Queiroz).

Indeed, the propensity for a severe COVID-19 epidemic in the neighborhoods of Fortaleza is very heterogeneous and reflects not only the population’s mobility in the urban space, but also the dynamics of transmission of a disease that is influenced by the living situation of a population in a city. Recently, an article indicating that the heterogeneity of the distribution of the incidence of COVID-19 is determined by socio-economic factors was published on the ABC American communication network. This article states that in New York City, a ‘stark contrast’ in COVID-19 infection rates can be observed, based on education and ethnicity [9].

The seven sociodemographic indicators assessed separately had spatial distributions with relevant heterogeneity. Inequality expressed by income (data not shown) had a distribution with an expression of less inequality in the periphery of the municipality and greater inequality in neighborhoods with better economic conditions, such as those in the eastern and coastal zones. Almost in a complementary way, the distribution of the proportion of unemployment showed higher rates in neighborhoods located in the outskirts of the municipality.

A similar situation was observed for indicators of household agglomeration (more than two people per bedroom) and households without access to water or sanitation. The inequality of these distributions indicates that these phenomena are correlated and probably express the evolution of the urban space occupation process in Fortaleza.

Very few studies have assessed the spread of the COVID-19 epidemic, and so far, no articles have been published that appreciat the influence of specific population factors linked to people’s mobility and to predict the occurrence of severe outbreaks in areas within cities. Spatial analysis was used by Kang et al. [10] to understand the epidemic spread of COVID-19. While the authors described the spatiotemporal pattern and evaluated the spatial association of the early stages of the COVID-19 epidemic in mainland China from January 16 to February 6, 2020, they sought only to identify the occurrence of spatial autocorrelation measured by Moran’s I for the various periods studied.

Fan et al. [11] studied the epidemiology of the Novel COVID-19 in Gansu Province, China. They concluded that different from findings from Wuhan Province, the spatial distribution pattern analysis indicated hot spots and spatial outliers in Gansu Province. To detect the spatial distribution pattern of COVID-19 cases at county levels during the study periods, they used local indicators of spatial association to evaluate the relationship between a given location and the surrounding spatial units by local Moran’s I (LISA).

Giuliani et al. [12] studied the spatiotemporal spread of COVID-19 in Italy. They sought to model and predict the number of COVID-19 infections, drawing out the effects of its spatial diffusion. They argue that “forecasts about where and when the disease will occur may be of great usefulness for public decision-makers, as they give the time to intervene on the local public health systems”. However, the authors did not consider the population heterogeneities and their influence in predicting the epidemic in the studied regions.

The study that used the methodological approach most similar to our study was conducted by Pluchino et al. [13]. They proposed a data-driven framework for assessing the epidemic risk of a geographical area (in a predictive way), and to identify high-risk areas within a country. They constructed a risk index combining three different features: (1) the disease hazard, (2) the infection exposure of the area, and (3) its vulnerability. However, vulnerability was considered based on the local data regarding air pollution, mobility, winter temperature, housing concentration, health care density, population size, and age.

Public transport is presented as a definite spatial trend, with trips mainly concentrated in the central and western regions of the city of Fortaleza, which are directly related to the provision of public transport and radio-concentric bus lines. However, as a part of the assumptions in this approved study, these dimensions should be considered while studying the transmission of SARS-CoV-2, in particular using samples of other respiratory-based infectious diseases [14, 15].

Our study aims to contribute to mathematical modelling studies in a complementary manner, in order to predict the dynamics of the COVID-19 epidemic in Brazil. Complementary methodological approaches are required to broaden the understanding of the epidemic and its possible determinants. Indeed, many mathematical models were used to estimate the epidemic curve of the COVID-19 outbreak in Brazilian cities. Rocha-Filho et al. [16] used a variant of the SEIR (Susceptible, Exposed, Infectious, Recovered) classical model, including hospitalized variables (SEIHR model) and an age-stratified structure to analyze the expected time evolution during the onset of the epidemic in the metropolitan area of São Paulo.

One of the main limitations of this study is that the prediction inherent to the methodological approach does not specify the time at which the severe epidemic will most intensely occur in the neighborhoods of Fortaleza. The simplicity of the approach used in our study, which does not exhaust all potential factors that influence the epidemic, is also an advantage over other methods.

## Conclusion

In conclusion, although universal actions should be applied to the entire municipality of Fortaleza, the classification of neighborhoods generated in this study can help improve specific measures in order to be more efficient. A set of recommendations were made to the municipal government of Fortaleza. The neighborhoods that were identified with a greater propensity for severe epidemics should receive special attention in the adoption of measures to control the epidemic.

## Supplementary information

**Additional file 1.** Letter of consent from the Municipal Health Secretariat of Fortaleza. Document referring to the consent of the Municipal Health Secretariat of Fortaleza secretariat giving the notification data aggregated by neighborhood of residence, which were published in the epidemiological bulletins and on the Fortaleza city hall website. In this document there is also reference about the need not submit the referred project to the ethics committee of that institution, nor of the other institutions.

## Data Availability

I confirm that the map in Figs. 1, 3, 4, 5, and 6. is my own. It was done with GIS software. The notification data for COVID-19 was obtained by request from the municipal health department of Fortaleza and the authors made a commitment to confidential use and confidentiality was guaranteed. Any researcher can request this data to the municipal health department of Fortaleza (https://www.fortaleza.ce.gov.br/institucional/a-secretaria-327) as long as the precepts contained in the data transfer rules of Brazilian law are met.

## Abbreviations

ESPIN: Public Health Emergency of National Importance
OD: Origin-Destination
HDU: Human Development Units
IPEA: Institute for Applied Economic Research
IBGE: Brazilian Institute of Geography and Statistics
LISA: Local Moran’s I
PEVI: Population epidemic vulnerability index
WHO: World Health Organization
