# Supplementary material for: Propensity for COVID-19 severe epidemic among the populations of the neighborhoods of Fortaleza, Brazil, in 2020
Source: BMC Public Health. 2020 Oct 1;20:1486. doi: 10.1186/s12889-020-09558-9 (PMC7527297; doi:10.1186/s12889-020-09558-9)
Supplement: Supplementary file 1 — Additional file 1. Letter of consent from the Municipal Health Secretariat of Fortaleza. Document referring to the consent of the Municipal Health Secretariat of Fortaleza secretariat giving the notification data aggregated by neighborhood of residence, which were published in the epidemiological bulletins and on the Fortaleza city hall website. In this document there is also reference about the need not submit the referred project to the ethics committee of that institution, nor of the other institutions. [file 12889_2020_9558_MOESM1_ESM.pdf]

Prefeitura de Fortaleza
Secretaria Municipal de Saúde

CARTA DE ANUENCIA

Informo que estou ciente da realização do estudo Propensão à epidemia grave de COVID-19 da população residente em bairros do Município de Fortaleza, 2020. Informo ainda que essa secretaria cedeu os dados de notificação agregados por bairro de residência, e que como são dados publicados nos boletins epidemiológicos e no site da prefeitura de Fortaleza, não foi necessário submeter o referido projeto ao comitê de ética dessa instituição, nem das demais instituições.

Atenciosamente,

Fortaleza, 15 de março de 2020.

Geziel dos Santos de Sousa
Secretaria Municipal de Saúde
